# Sympathetic Effect of Auricular Transcutaneous Vagus Nerve Stimulation on Healthy Subjects: A Crossover Controlled Clinical Trial Comparing Vagally Mediated and Active Control Stimulation Using Microneurography

**DOI:** 10.3389/fphys.2020.599896

**Published:** 2020-12-03

**Authors:** Anaïs Gauthey, Sofia Morra, Philippe van de Borne, Denis Deriaz, Nathalie Maes, Jean-Benoît le Polain de Waroux

**Affiliations:** ^1^Department of Cardiology, Saint-Luc Hospital, Université catholique de Louvain, Brussels, Belgium; ^2^Department of Cardiology, Erasme Hospital, Université libre de Bruxelles, Brussels, Belgium; ^3^Department of Biomedical and Preclinical Sciences, Université de Liège, Liège, Belgium; ^4^Department of Biostatistic and Medico-Economic Information, CHU Hospital of Liège, Liège, Belgium; ^5^Department of Cardiology, AZ Sint Jan, Bruges, Belgium

**Keywords:** cardiac autonomic function, healthy subjects, muscle sympathetic nerve activity (MSNA), auricular transcutaneous vagus nerve stimulation, auricular branch of the vagus nerve

## Abstract

Introduction: Auricular low-level transcutaneous vagus nerve stimulation (aLL-tVNS) has emerged as a promising technology for cardiac arrhythmia management but is still experimental. In this physiological study, we hypothesized that aLL-tVNS modulated the autonomic nervous balance through a reduction of sympathetic tone and an increase in heart rate variability (HRV). We investigated the muscle sympathetic nerve activity (MSNA) recorded by microneurography during vagally mediated aLL-tVNS and active control on healthy volunteers. Methods: In this crossover, double-blind controlled study, healthy men (*N* = 28; 27 ± 4 years old) were assigned to aLL-tVNS applied to cymba and lobe (active control) of the right ear. Each participant was randomly allocated to the three sequences (5 Hz, 20 Hz, and active control-5 Hz) during one session. MSNA signal was recorded at rest, during voluntarily apnea and aLL-tVNS. Sympathetic activity was expressed as: 1) number of bursts per minute (burst frequency, BF) and 2) MSNA activity calculated as BF x mean burst amplitude and expressed as changes from baseline (%). RR intervals, HRV parameters and sympathetic activity were analyzed during 5 min-baseline, 10 min-stimulation, and 10 min-recovery periods. Mixed regression models were performed to evaluate cymba-(5—20 Hz) effects on the parameters with stimulation. Results: During apnea and compared to baseline, BF and MSNA activity increased (*p* = 0.002, *p* = 0.001, respectively). No stimulation effect on RR intervals and HRV parameters were showed excepted a slightly increase of the LF/HF ratio with stimulation in the cymba-5Hz sequence (coef. ± SE: 0.76 ± 0.32%; *p* = 0.02). During stimulation, reductions from baseline in BF (Coef. ± SE: −4.8 ± 1.1, *p* < 0.001) was observed but was not statistically different from that one in the active control. Reduction of MSNA activity was not significantly different between sequences. Conclusion: Acute right cymba aLL-tVNS did not induce any overall effects neither on heart rate, HRV nor MSNA variables on healthy subjects when compared to active control. Interestingly, these findings questioned the role of active controls in medical device clinical trials that implied subjective endpoints.

## Introduction

Cardiac autonomic disbalance represents a prerogative for the onset and maintenance of atrial fibrillation (AF) (Bettoni and Zimmermann, 2002; Chen et al., 2014; Stavrakis et al., 2015b; Agarwal et al., 2017). Atrial ganglionated plexus ablation in addition or not to pulmonary veins (PV) isolation, has demonstrated a significant benefit for free-recurrence of AF (Katritsis et al., 2013; Stavrakis and Po, 2017). Auricular low level transcutaneous vagus nerve stimulation (aLL-tVNS) has been described to inhibit ganglionated plexus and stellate activities. Cholinergic anti-inflammatory pathway mediated by tVNS involves a reduction in pro-inflammatory cytokines levels (Stavrakis et al., 2015a). Also, aLL-tVNS increases atrial and PV myocardial refractory periods (Yu et al., 2013; Stavrakis et al., 2015a). Concordantly, this technique is susceptible to modulate both the trigger and substrate for AF along with autonomic, electrical and structural atrial remodelings (Zhu et al., 2019; Stavrakis et al., 2020). Nevertheless, physiological effects on sympathovagal balance remain to be fully understood before standardizing the use of tVNS devices for clinical applications. Feasibility of aLL-tVNS as a reliable alternative to invasive cervical VNS is driven by the cutaneous distribution of vagal fibers through its auricular branch (ABVN) and its subsequent afferents projections illustrated by functional magnetic resonance imaging (fMRI) (Frangos et al., 2015; Badran et al., 2018a; Butt et al., 2019). Published randomized and non-randomized aLL-tVNS studies on cardiovascular effects in healthy subjects (Clancy et al., 2014; Butt et al., 2019) are attractive but would require to be validated by controlled designs with direct assessment of orthosympathetic activity. Muscle sympathetic nerve activity (MSNA) assessed by the microneurography, records directly the sympathetic activity directed toward peripheral blood vessels while analysis of heart rate variability (HRV) indirectly reflects changes in cardiac parasympathetic activity (Heart rate variability., 1996; David, 2011; Iwase et al., 2017). As potential explanation for aLL-tVNS mechanisms, excitatory signals from afferents vagal fibers to the nucleus of the solitary tract (NTS) and caudal ventrolateral medulla (CVLM) would result in both a reduction and an increase in sympathetic and parasympathetic tones, respectively (Clancy et al., 2014). Inhibitory signals from CVLM to the rostro ventrolateral medulla (RVLM), well described as the mainstay of sympathetic output sent inhibitory signals to the sympathetic paravertebral ganglionic chain resulting in a decrease in sympathetic activity (Murray et al., 2016). In this crossover, double-blind controlled study, we focused on acute and direct effects of aLL-tVNS on sympathetic tone. As MSNA directly assesses the sympathetic ganglionic neuron activity (Guyenet, 2006), we hypothesized that aLL-tVNS lowered the sympathetic activity measured by microneurography. For this purpose, we investigated the MSNA signal recorded by microneurography during cymba aLL-tVNS and active control on healthy volunteers.

## Materials and Methods

### Study Design

This was a clinical experimental, double-blind with crossover-controlled study. All the participants were tested for three sequences: cymba-(5—20 Hz) stimulation along with active control (earlobe-5 Hz) in a simple randomly allocated order. Prior to each session, randomness of the assignment to one of the sequences was determined throwing a dice. Thus, there were in total six possible combinations. The random list for the sequences (one number of the dice corresponding to one combination of sequences) has been established prior to the first participant session. Each phase (baseline, stimulation and recovery times) of each sequence (cymba-5 Hz; cymba-20 Hz, and earlobe-5 Hz) for all participants dataset received a random allocated alphanumerical code so that operators were blinded for the inspection of the neurogram. Statistical analyzes were fully blinded to the operators and performed by the biostatisticians (DD and NM).

### Participants and Data

Healthy, young and active men (27 ± 4 years old) were enrolled (*N* = 28) from June 2019 until November 2019 in one single center by the two investigators. Only male gender was included based on the following considerations: 1) avoiding confounding contribution of menstrual cycle on the measurements; 2) easier identification of peroneal in men probably due to lower amount of subcutaneous fat (Stephens and Kelly, 2000); and 3) lower resting MSNA activity reported in women (Matsukawa et al., 1998). Candidates were eligible if they did not have any cardiovascular nor neurological nor mental diseases and if they did not take any medication. They had to be over 18 years of age and they were asked to avoid intense exercise and alcohol, and were asked to refrain from smoking and taking caffeine the day before participation. Prior to the experimental session, participants were asked to empty their bladder. Our local ethics committee (P2019/264; 2017/14JUI/317) approved the study and all patients consented orally and in written to participate to the study.

HRV analyzes were available for all the subjects (*N* = 28). Successful recording rate for MSNA signal identification was 64% (*N* = 18/28) with a good signal to noise ratio (5 Hz: *N* = 16/18; 20 Hz: *N* = 15/18).

### Intervention

**FIGURE 1:**
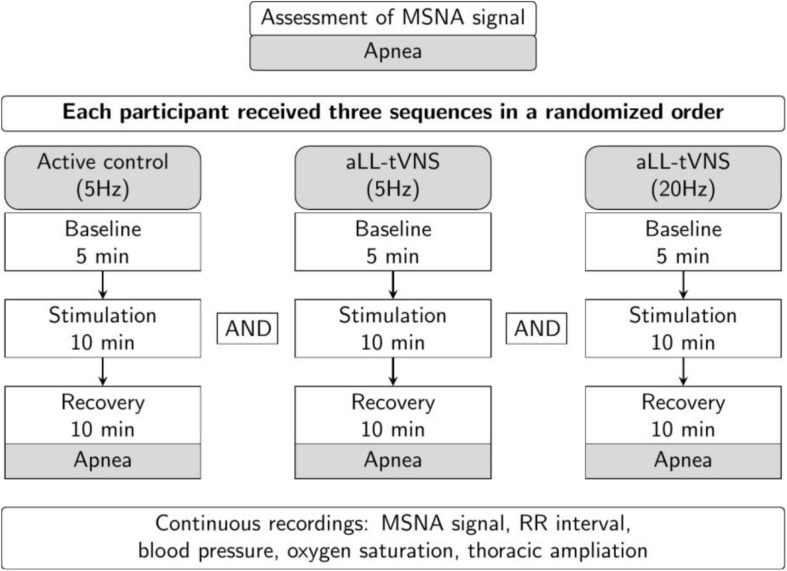
Study design. Consecutive sequences (active control-5 Hz, aLL-tVNS-5 Hz; aLL-tVNS-20 Hz) were randomly ordered and composed of 5 min-baseline, 10 min-stimulation, and 10 min-recovery phases. Apnea were performed before and after each sequence.

**FIGURE 2:**
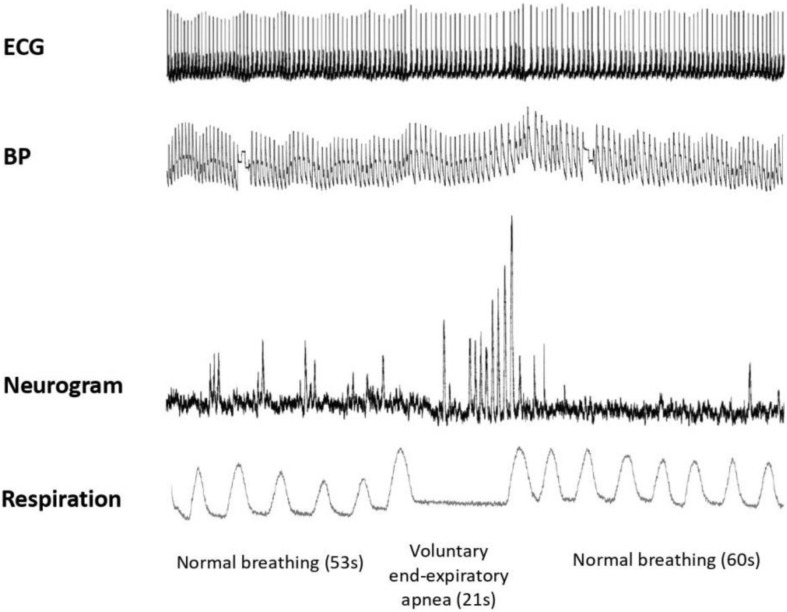
MSNA variations during baseline and voluntary end-expiratory apnea. Normal respiration during baseline (53 s length), maximal voluntary end-expiratory apnea (21 s length), and normal respiration during recovery (60 s) are illustrated. From top to bottom: ECG; blood pressure (BP); neurogram and respiration. Toward the end of the apnea, a marked rise in the sympathetic nerve activity is observed, characterized by increased number of bursts with higher amplitude.

First, we assessed the MSNA using the microneurography technique. Participants underwent subsequent aLL-tVNS either performed on the cymba of the right ear applying low frequency (5 Hz) and high frequency (20 Hz) stimulation and on the lobe of the right ear applying low frequency (5 Hz) in a randomly order. They were blinded for assignment. Each sequence included a 5 min-baseline followed by a 10 min-stimulation and a 10 min-recovery (wash-out) phases along with continuous MSNA recording (Figure 1). During the session and in between all the three sequences, the adequacy of the nerve recording site was acertained by means of voluntary end-expiratory sustained apnea performed by participants with a subsequent increase in the MSNA activity (Figure 2). The duration of the apnea differed between individuals; each of them was required to hold his breath as long as he could (from a minimum of 13 s to a maximum of 49 s). The aim of this maneuver was to induce modification of blood gases concentration to activate the MSNA and to make sure the needle was correctly placed, and the obtained record coincided with MSNA activity and not with SSNA one. The identification of the apnea was based on the respiratory signal measured with the respiratory belt: the beginning of the apnea was identified at the end of a maximal expiration, the lungs being at their residual volume, the end of the apnea was identified prior to the restauration of respiration.

Sample size calculation was limited by the lack of reliable and objective effect size for physiological tVNS impact either on cardiac autonomic function (HRV markers) and peripheral sympathetic tone (MSNA). Several considerations could participate to the varied tVNS response reported in the literature: 1) the inter-subject variability could limit the reproducibility of the results along with 2) the heterogeneity among tVNS protocols (setting parameters); 3) the need for more tVNS studies evaluating the sympathetic markers such as MSNA; and 4) the complexity of the autonomic nervous system and clinical covariates that limited the evaluation of a one size effect-parameter. Nonetheless, we performed this study using a crossover design with each subject being its own control, requiring a smaller cohort to achieve outcomes and allowing for precise description of the intervention effect.

### Statistical Methods

Outcomes measured were: 1) direct comparison of the changes in RR intervals, HRV, blood pressure and MSNA parameters between cymba-aLL-tVNS (5—20 Hz) and active control sequence (earlobe-5 Hz); 2) correlation between the variation of MSNA activity (%baseline) and RMSSD, SDRR, or the LF/HF ratio.

Distribution of continuous variables were graphically checked at each phase level for HRV variables and at minute level for both BP and MSNA variables. Both outliers’ observations (*N* = 3) and artifacts (0.78% of the pooled MSNA recordings) were dropped out for statistical analysis. Continuous variables were expressed as means and standard deviations (mean ± SD) and categorical variables were reported as counts and proportions. SDRR (ms), LF and HF components (ms2) and MSNA (AU/s) were log-transformed. Mixed linear regression models were used to analyze the stimulation effect on the evolution of variables with stimulation.

For each variable, models included the sequence effect (active control-5 Hz = reference, cymba-5 Hz, and cymba-20 Hz), the stimulation effect (baseline = reference, and stimulation), and the stimulation × sequence effect. Random subject intercepts were added to the models in order to take into account the subject-specific variation. For the active control-5 Hz, cymba-5 Hz, and cymba-20 Hz stimulation, effect delay was tested by dividing the stimulation phase in two 5 min subphases. Pearson correlations between the variation of MSNA activity (%baseline) and RMSSD, SDRR, or the LF/HF ratio were also computed. Data analysis was carried out using R software (version 3.6.2) and SAS software (version 9.4) and results were considered significant at the 5% critical level (*p* < 0.05).

### Transcutaneous Vagus Nerve Stimulation

Low frequency (5 Hz) and high frequency (20 Hz) stimulation with a fixed pulse duration of 0.2 ms were delivered using a tVNS system dedicated to target the right cymba and the earlobe. Earlobe, currently used as the standard not vagally mediated site in fMRI studies (Kraus et al., 2007, 2013; Frangos et al., 2015; Yakunina et al., 2017; Badran et al., 2018a) was considered for active control sequence (Butt et al., 2019). Subject’s right ear was cleaned and dried so that a good contact between earpiece and skin was ensured. Individually intensity level (mA) was based on sensory perception (De Couck et al., 2017; Borges et al., 2019).

### Microneurography

Firstly used in the mid 60’s to record action potentials on peripheral human nerves, microneurography directly assesses the efferent sympathetic activity directed to vascular smooth muscle of vessels called MSNA (Delius et al., 1972; Vallbo, 2018). The equipment was composed of: two tungsten needle electrodes (μm), an amplifier to increase the raw signal and improved signal to noise ratio, a signal integrator (GRASS, Instrumental division, Astro-Med^®^) and an output (computer software-ADInstrument) (White et al., 2015). Subjects were tested at rest in a semi-supine position with a pillow under the head and a supported pillow under the right leg so that the site of stimulation remained stable for all the experiment. The experimental session was realized in a dedicated room with temperature controlled.

First part of the session consisted in peroneal nerve of the right leg identification by cutaneous electrical stimulation (Vallbo et al., 1979, 2004; White et al., 2015). Then, an active micro electrode (UNA35F2S, FHC Neural MicroTargeting^TM^) was inserted into the peroneal nerve which was preferentially choosen to record MSNA because of its easy accessibility. The reference electrode was placed in the subcutaneous tissue 2–3 cm away from the active one. Electrode adjustements and audiomonitoring were made until a clear MSNA signal was achieved. Establishement of the MSNA signal was assessed on real time using the following criteria: 1) diastolic-pulsed-synchronized, 2) no influence of startle nor sensory stimuli, and 3) respiratory modulated (Iwase et al., 2017). Raw signal was processed to be amplified, filtered, integrated and connected to the acquisition system PowerLab 16/30 (ADInstruments). To avoid the unwanted noise of electrical stimulus from 5 to 20 Hz frequency stimulations, an automatic band-pass filter was applied. Each burst was manually identified by a trained operator. The amplitude of each burst was determined (arbitrary units, AU). Required amplitude of the normalized signal had to be at least a 2:1 signal to noise ratio, as previously described (White et al., 2015). Burst frequency (number of burst/min), burst incidence (BI; bursts/100 heart beats) and MSNA activity were reported. Burst amplitude varied along with amplification and nerve position among subjects. MSNA activity was calculated as burst frequency multiplied by mean burst amplitude (AU) expressed in percentage from baseline value to allow inter-participant comparison (Najem et al., 2006).

### Others Data Acquisition

One lead-ECG, systemic blood pressure, oxygen saturation and respiratory ampliation signals were continuously assessed during the experimental session (Figure 1). Breathing was free (17 ± 3 breath/min). Prior to HRV analysis, adequate R peak detection was manually checked. Beat-to-beat RR interval analysis was automatically processed using the HRV module for LabChart Pro v8 (ADInstruments) after exclusion of ectopic beats. A 1,000-Hz sampling frequency was set by default for HRV analysis.

Standard deviation of RR intervals (SDRR) and root mean square of the successive RR interval differences (RMSSD) were used for time-domain analysis. Low (LF), high (HF) frequency power, and LF/HF ratio were used for frequency-domain measurements (Iwase et al., 2017; Shaffer and Ginsberg, 2017). Beat-to-beat systemic blood pressure was acquired by a finger cuff (Finometer Pro, FMS©, Amsterdam, Netherlands) and analyzed off-line through the Blood Pressure Module for LabChart (ADInstruments). Brachial blood pressure was measured using an automatic manometer to confirm finometer values. Oxygen saturation and respiratory ampliation signal were obtained using a pulse oximeter (Capnostream-35-monitor ©, Oridion Medical 272 Ltd., Jerusalem, Israël) and a chest belt (ADInstruments), respectively.

## Results

All participants (*N* = 28) were healthy young men with baseline characteristics reported in Table 1. Mean stimulation intensities at cymba-(5—20 Hz) and active control-5 Hz were 1.5 ± 0.6, 1.2 ± 0.5, and 5.5 ± 1.6 mA, respectively, which is concordant with others studies (De Couck et al., 2017; Yakunina et al., 2017; Borges et al., 2019). During apnea and compared to baseline, burst frequency and MSNA activity increased (*p* = 0.002; *p* = 0.001, respectively) which specifically featured effective sympathetic tone modulation in response to breathing cessation. At the beginning of the apnea, there is a suppression of the sympathetic nerve activity without discernable bursts. HR accelerates compared to normal respiration and systolic blood pressure slightly falls. Toward the end of the apnea, a marked rise in the sympathetic nerve activity is observed, characterized by an increase of BF and MSNA. HR slows and systolic blood pressure rises compared to the beginning of the apnea (Figure 2).

**TABLE 1:** Participants baseline characteristics.

**Variable**	**Mean ± SD or *n* (%)**
Male, *n* (%)	28 (100%)
Age (years)	27 ± 4
BMI (kg/m^2^) ** Hemodynamics**	23.5 ± 3.2
SBP (mmHg)	126 ± 10
DBP (mmHg)	72 ± 6
MAP (mmHg) Respiratory rate (breath/min) HR (bpm)	90 ± 7 17 ± 3 64 ± 9
**Intensity (mA)**	
Active control-5 Hz aLL_tVNS-5 Hz aLL-tVNS-20 Hz	1.5 ± 0.6 1.2 ± 0.5 5.5 ± 1.6

### Stimulation Effects of the 3 Sequences on Parameters

#### Effects of aLL-tVNS on Heart Rate Variability

**FIGURE 3:**
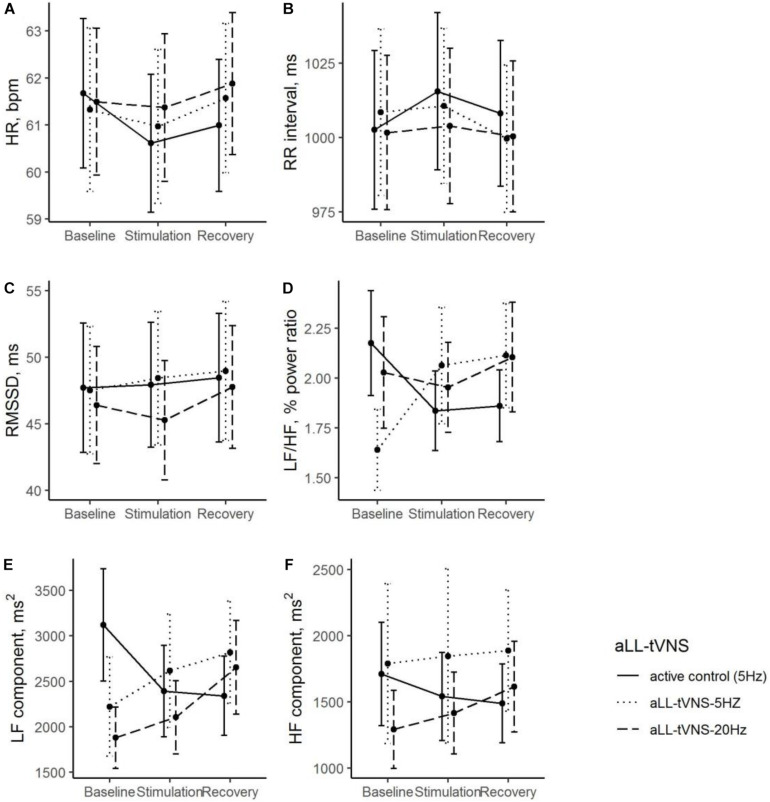
During all the consecutive aLL-tVNS sequences (active control-5 Hz, aLL-tVNS-5 Hz, aLL-tVNS-20 Hz), evolution (mean ± SD) of heart rate **(A)**, RR interval **(B)**, RMSSD **(C)**, LF/HF ratio **(D)**, LF **(E)**, and HF **(F)** components per phase (baseline, stimulation, and recovery). No stimulation effect on RR intervals and HRV parameters were showed excepted a slightly increase of the LF/HF ratio with stimulation in the cymba-5 Hz sequence (coef. ± SE: 0.76 ± 0.32; *p* = 0.02).

No overall stimulation effect on RR intervals nor HRV parameters was demonstrated excepted in the LF/HF ratio (cymba-5 Hz). LF/HF ratio was significantly lower in the cymba-5 Hz (Coef. ± SE: −0.54 ± 0.23, *p* = 0.021) and increased significantly with stimulation in this sequence (Coef. ± SE: 0.76 ± 0.32; *p* = 0.020). HF was significantly lower in the cymba-20 Hz sequence and no stimulation effect was noted. LF was significantly lower in the cymba-(5—20 Hz) sequences and no stimulation effect was noted (Table 2 and Figure 3). No effect delay during stimulation was observed (*p*-values >0.05 for all parameters).

**TABLE 2:** Study of aLL-tVNS effects (linear mixed model).

	**Independent variable**	**Coefficient ± SE**	***p*-value**
HR, bpm	Intercept	61.7 ± 1.6	–
	5-Hz stimulation sequence (ref = control)	−0.3 ± 0.6	0.57
	20-Hz stimulation sequence (ref = control)	−0.2 ± 0.6	0.77
	Stimulation phase (ref = baseline phase)	−1.1 ± 0.6	0.09
	5-Hz stim sequence × Stim phase	0.7 ± 0.9	0.42
	20-Hz stim sequence × Stim phase	0.9 ± 0.9	0.28
RMSSD, ms	Intercept	47.7 ± 4.7	–
	5-Hz stimulation sequence (ref = control)	−0.2 ± 2.1	0.93
	20-Hz stimulation sequence (ref = control)	−1.3 ± 2.1	0.53
	Stimulation phase (ref = baseline phase)	0.2 ± 2.1	0.91
	5-Hz stim sequence × Stim phase	0.7 ± 2.9	0.82
	20-Hz stim sequence × Stim phase	−1.4 ± 2.9	0.64
SDRR, ms	Intercept	70.5 ± 4.9	-
	5-Hz stimulation sequence (ref = control)	−**7.2 ± 3.0**	**0.020**
	20-Hz stimulation sequence (ref = control)	−**6.2 ± 3.0**	**0.043**
	Stimulation phase (ref = baseline phase)	−3.1 ± 3.0	0.31
	5-Hz stim sequence × Stim phase	6.7 ± 4.3	0.12
	20-Hz stim sequence × Stim phase	1.0 ± 4.3	0.81
LF component,	Intercept	7.41 ± 0.21	–
ms^2^*	5-Hz stimulation sequence (ref = control)	−**0.33 ± 0.16**	**0.036**
	20-Hz stimulation sequence (ref = control)	−**0.31 ± 0.16**	**0.048**
	Stimulation phase (ref = baseline phase)	−0.11 ± 0.16	0.48
	5-Hz stim sequence × Stim phase	0.27 ± 0.22	0.23
	20-Hz stim sequence × Stim phase	0.17 ± 0.22	0.44
HF component,	Intercept	6.86 ± 0.21	–
ms^2^*	5-Hz stimulation sequence (ref = control)	−0.08 ± 0.11	0.48
	20-Hz stimulation sequence (ref = control)	−**0.25 ± 0.11**	**0.025**
	Stimulation phase (ref = baseline phase)	−0.01 ± 0.11	0.92
	5-Hz stim sequence × Stim phase	−0.002 ± 0.153	0.99
	20-Hz stim sequence × Stim phase	−0.09 ± 0.15	0.56
LF/HF ratio,	Intercept	2.18 ± 0.25	–
% power ratio	5-Hz stimulation sequence (ref = control)	−**0.54 ± 0.23**	**0.021**
	20-Hz stimulation sequence (ref = control)	−0.15 ± 0.23	0.52
	Stimulation phase (ref = baseline phase)	−0.34 ± 0.23	0.14
	5-Hz stim sequence × Stim phase	**0.76 ± 0.32**	**0.020**
	20-Hz stim sequence × Stim phase	0.26 ± 0.32	0.42
SBP, mmHg	Intercept	114.7 ± 2.0	–
	5-Hz stimulation sequence (ref = control)	1.0 ± 1.1	0.39
	20-Hz stimulation sequence (ref = control)	−0.1 ± 1.1	0.93
	Stimulation phase (ref = baseline phase)	0.2 ± 1.1	0.87
	5-Hz stim sequence × Stim phase	−1.3 ± 1.6	0.42
	20-Hz stim sequence × Stim phase	−0.3 ± 1.6	0.86
DBP, mmHg	Intercept	58.5 ± 1.7	–
	5-Hz stimulation sequence (ref = control)	0.5 ± 0.7	0.47
	20-Hz stimulation sequence (ref = control)	0.3 ± 1.7	0.64
	Stimulation phase (ref = baseline phase)	−0.1 ± 0.7	0.89
	5-Hz stim sequence × Stim phase	−0.3 ± 1.0	0.75
	20-Hz stim sequence × Stim phase	0.2 ± 1.0	0.84
MBP, mmHg	Intercept	77.2 ± 1.7	–
	5-Hz stimulation sequence (ref = control)	0.6 ± 0.8	0.41
	20-Hz stimulation sequence (ref = control)	0.2 ± 0.8	0.82
	Stimulation phase (ref = baseline phase)	0.0 ± 0.8	0.99
	5-Hz stim sequence × Stim phase	−0.6 ± 1.1	0.57
	20-Hz stim sequence × Stim phase	0.0 ± 1.1	0.97
BF, bursts per	Intercept	17.6 ± 2.7	–
minute	5-Hz stimulation sequence (ref = control)	1.4 ± 1.1	0.21
	20-Hz stimulation sequence (ref = control)	−1.5 ± 1.1	0.19
	Stimulation phase (ref = baseline phase)	−**4.8 ± 1.1**	**<0.001**
	5-Hz stim sequence × Stim phase	0.5 ± 1.6	0.75
	20-Hz stim sequence × Stim phase	1.3 ± 1.6	**0.41**
MSNA activity,	Intercept	3.95 ± 0.20	–
AU/min*	5-Hz stimulation sequence (ref = control)	0.12 ± 0.10	0.21
	20-Hz stimulation sequence (ref = control)	−0.09 ± 0.10	0.34
	Stimulation phase (ref = baseline phase)	−0.13 ± 0.10	0.19
	5-Hz stim sequence × Stim phase	−0.06 ± 0.14	0.66
	20-Hz stim sequence × Stim phase	−0.003 ± 0.139	0.99

*Models are predicting the log-transformed variable. Significant values at level α = 5% are bolded.

#### Effects of aLL-tVNS on Blood Pressure Parameters

No stimulation effect was observed for all the blood pressure variables (Table 2).

#### Effects of aLL-tVNS on MSNA Parameters

**FIGURE 4:**
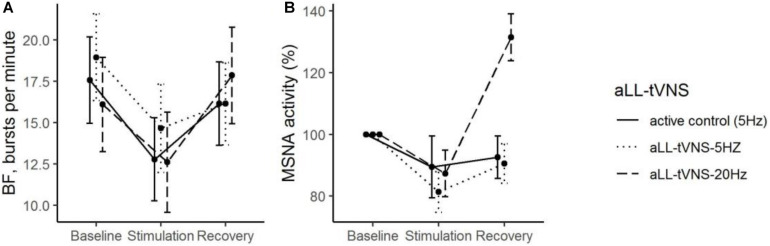
During all the consecutive aLL-tVNS sequences (active control-5 Hz, aLL-tVNS-5 Hz, and aLL-tVNS-20 Hz), evolution (mean ± SD) of burst frequency **(A)** and MSNA activity (%) **(B)** per phase (baseline, stimulation, and recovery). During stimulation, reductions from baseline in BF (Coef. ± SE: –4.8 ± 1.1, *p* < 0.001) was observed but was not statistically different from that one in the active control. Reduction of MSNA activity was not significantly different between sequences.

During stimulation, reductions from baseline in BF (Coef. ± SE: −4.8 ± 1.1, *p* < 0.001) was observed. However, this evolution was not statistically different from that one in the active control (Table 2 and Figure 4). Reduction of MSNA activity was not statistically significant (Table 2). We did not find any correlation between MSNA activity and HRV parameters.

For additional information regarding descriptive statistics, see Supplementary Table 1 (additional Table 1).

## Discussion

### Results Summary

To the best of our knowledge, our study is the first to explore the specific aLL-tVNS effects on sympathetic tone using microneurography compared to active control in crossover trial with randomly allocated stimulation sequences. Our results did not demonstrate any overall effects of cymba aLL-tVNS neither on HRV nor MSNA variables compared to active control (earlobe sequence-5 Hz). Interestingly, an active control response may also be suggested.

### Heterogeneous Response of Autonomic Nervous System to Stimuli

HRV analysis is currently used for clinical assessment of cardiac autonomic function with the assumptions that LF and HF power reflect sympathetic and vagal modulation respectively. Also, the LF/HF ratio has been suggested as an index for sympathovagal balance (Heart rate variability., 1996; Iwase et al., 2017). Obviously, interpretation of those HRV parameters remains questionable since they are indirect indexes of the autonomic balance at the cardiac level. For each of them, LF and HF components resulted in a mix and variable proportion of orthosympathetic (ONS) and parasympathetic (PNS) systems (Iwase et al., 2017; Shaffer and Ginsberg, 2017). Same LF/HF ratio values could refer to different fluctuations of PNS or ONS or both of them. Also, varying effects between the two systems should not be considered as reciprocal. Indeed, heterogeneous response like “diving reflex” with observed bradycardia along with activated ONS clearly indicated the complexity of the relationship between the two pathways (Gooden, 1994). Therefore the clinical significance of LF/HF ratio has not been yet fully clarified (Billman, 2013).

LF/HF ratio has been shown to be reduced by aLL-tVNS which is associated with improved HRV in healthy humans (Clancy et al., 2014). In contrast, in the present study, we identified a slightly increase in the LF/HF ratio during the cymba-5 Hz stimulation compared to active control (earlobe-5 Hz) suggesting a potential shift toward a sympathetic predominance. However, an important remark concerned the context which remained crucial for HRV measurements interpretation:1) LF/HF ratio was significantly lower (cymba-5Hz) compared to active control (Table 2 and Figure 3) and 2) a more sensitive perception of the stimulus when applied to cymba versus ear lobe could participate to explain the observed shift in the autonomic balance. Therefore, the clinical conclusion relative to this result may be not relevant. Further and for all the three sequences, no correlation was found between HRV parameters and MSNA activity highlighting the complexity to accurately interpret those variations mediated by two different systems (autonomic cardiac and peripheral sympathetic activities). Noteworthily, aLL-tVNS effects on HRV are hard to carry out since mixed results exist in the literature probably due to the variety of study designs, control groups, protocols and stimulation parameters, etc. Finally, although our results did not demonstrate any consistent aLL-tVNS effects on HRV, they provide substantial feedback on ONS response to aLL-tVNS using microneurography.

### Variability Among aLL-tVNS Studies on Cardiac Autonomic System

Compared to Clancy et al. (2014), we report higher baseline values for spectral HRV parameters. However, our participants were younger, healthy and exclusively active male. These characteristics are well known to be associated with LF power and SDRR (Berkoff et al., 2007; De Couck et al., 2017; Shaffer and Ginsberg, 2017). Variability among aLL-tVNS parameters (frequencies, site of stimulation and intensity levels, etc.) could also play a role in the heterogeneity of the results observed in the literature. In their elegant review Butt et al. (2019), summarized the different settings and findings of aLL-tVNS studies focusing on cardiovascular parameters among healthy subjects. As underscored by the authors, the various results observed on HRV could be related to differences among the stimulation protocols (Clancy et al., 2014; De Couck et al., 2017). Beyond parameters, the location of auricular stimulation sites also differs between the different studies. We used a device designed to target the cymba as this region is exclusively innervated by ABVN with a more expected impact on HRV and strong evidence of vagal activated projections (Groves and Brown, 2005; De Couck et al., 2017; Yakunina et al., 2017). In contrast, others used tragus-dedicated systems (Clancy et al., 2014; Stavrakis et al., 2015a). With the right vagus nerve destinated to the sinoatrial node and the left one dedicated to atrioventricular node, the stimulation side is also a potential source of discrepancy. As we wanted to specifically explore effects on HRV, we decided to target the right cymba (De Couck et al., 2017). Control group also varies with either inactive or active aLL-tVNS (Kraus et al., 2007; Clancy et al., 2014; De Couck et al., 2017). Also, stimulation settings (intensity levels, pulse width, frequency, duration of the stimulus, etc.) ranged widely. As De Couck et al. (2017), we used a personalized thresholding to define intensity level but others refer to a “set stimulation method” in which intensity was determined by the operator (Borges et al., 2019). Two frequencies of stimulation (5 and 20 Hz) were tested in our study based on the following considerations. Vagus nerve is composed of afferent (80%) and efferent (20%) fibers with a majority of C fibers activated at low frequency stimulation (5 Hz) and high intensity level (mA) (Groves and Brown, 2005; Yu et al., 2013). Nonetheless, fibers nerve composition differs between vagus nerve and ABVN, the latter containing more A fibers themselves activated at higher frequency (20 Hz) (Safi et al., 2016). But others have recommended the frequency of 10 Hz (pulse width: 500 μs) for its major reduction of heart rate (Badran et al., 2018b). Several explanations for the lack of differences during stimulation between sequences could be suggested: 1) an active control response to aLL-tVNS cannot be excluded. The latter also questioned the implication of subjective endpoints to explain the observed results; 2) our healthy and active population may have challenged the modulation of a “normal” autonomic state; 3) settings parameters might have been not optimal to activate afferent vagal pathway but higher intensities would have led to discomfort; and 4) the small number of participants could have limited the results.

### Active Control Response of aLL-tVNS

Using the crossover design with active control stimulation performed on the earlobe, we observed a signification reduction in BF during stimulation, but this was not related neither to stimulation frequency nor to site of stimulation. Strongly supported by an accurate modulation of ONS illustrated by MSNA changes during apnea, these results are in favor of a sympathetic mediated effect. We did not identify any predictors for the active control response. Validating an effect during the active control sequence would have implied a direct comparison with a “no treatment” sequence (Enck et al., 2011). Nonetheless, taking into account that our study was initially designed to compare cymba aLL-tVNS versus ear lobe aLL-tVNS, we may questioned the subjective outcomes in the observed results. Some relevant points could be pointed out: 1) active control with crossover along with MSNA protocols are lacking. Indeed, active control aLL-tVNS is not systematic (Clancy et al., 2014; De Couck et al., 2017) as well as MSNA recording (Antonino et al., 2017; Borges et al., 2019). Published aLL-tVNS studies on cardiovascular parameters are promising but comparison with active control stimulation would help to validate the effectiveness of the therapy. Using a crossover design with each subject being its own control, confounding variates influences are limited. From statistical considerations, this allows for smaller sample size. As sequences were randomized, order effect is excluded. We checked if the results were robust to the order of sequence attribution adding a categorical variable to the regression models. Also, each sequence was composed of a baseline and recovery periods which has the advantage to manage carry-over effects. aLL-tVNS studies conventionally explored the potential effect of aLL-tVNS compared to control but not in the reverse way. 2) Ear lobe as a reliable site for active control stimulation (Kraus et al., 2007; Frangos et al., 2015; Badran et al., 2018a) may be questioned despite its innervation free from vagal fibers (Peuker and Filler, 2002). Indeed, changes in BOLD signal induced by ear lobe transcutaneous stimulation in healthy subjects has been documented through fMRI studies (Butt et al., 2019). Published activation brain maps for several ear location stimulation, highlighted that earlobe projections overlap with cymba projections for some cortical areas. However, NTS nor locus coeruleus, two major targets for tVNS mechanisms were concerned by this crossing (Frangos et al., 2015; Yakunina et al., 2017). Ear lobe is definitively not physiologically inert challenging tVNS methodology in clinical trials. We could mention cymba as ABVN dedicated region to be used for both active and control stimulation but with different settings (Bauer et al., 2016).

### Active Control Design for aLL-tVNS Medical Devices

As the present work was designed to demonstrate a specific effect of tVNS on autonomic balance, we included an active control sequence (earlobe-5 Hz) not vagally mediated and a crossover design as a control strategy. We questioned here the role of controls in clinical trials with non-invasive medical devices like tVNS that implied subjective endpoints. This should be integrated to methodology to test the efficacy of tVNS itself that would not be reliable to a relaxation state of the subjects. Question may be clinically relevant as well as feasibility with no additional risk making that active control ethically acceptable (Sutherland, 2007). Effectiveness of aLL-tVNS devices should clearly be distinguished from active control effect/subjective outcomes before approval and commercial use of the technique (Redberg, 2014). The importance of controlled design certainly made sense to improve the understandings of aLL-tVNS mechanisms so that optimal parameters of stimulation could be defined.

## Limitations

This study has several limitations. First, the small number of participants might have been insufficient to demonstrate aLL-tVNS impact on sympathetic tone (type II error) but this had to be integrated with the technical challenge of MSNA acquisition. Second, carryover effect could have not been excluded despite the wash out period of 10 min whom duration was limited by the required stability of the active needle inserted into the peroneal nerve around subdermal tissues to obtain high quality signal of MSNA.

We observed a response during active control sequence, but this could be questioned, as active control may not be free from specific effect. Although study design was focused on active aLL-tVNS rather than on control effect, we discussed this with limited bias using a crossover-controlled design. Second, all our subjects were healthy and young men so no extrapolation could be made for other groups (women, elderly, etc.) and particularly for patients with cardiac arrhythmia. Third, despite prior cleaning of the ear, skin properties could have limited the delivery of the electrical signal. Also, even if participant were asked to breath constantly, respiration rate fluctuations could have influenced HRV parameters. Finally, active maneuvers such as standing and/or tilt test could have been an alternative to evaluate the effects of aLL-tVNS on autonomic balance.

## Conclusion

Acute right cymba aLL-tVNS did not induce overall effects neither on heart rate, HRV nor MSNA variables on healthy subjects compared to active control. These findings questioned the role of active controls in medical device clinical trials that implied subjective endpoints.

## Data Availability Statement

Data can be provided by authors upon reasonable request.

## Ethics Statement

The studies involving human participants were reviewed and approved by Ethics Committee Hospitalo-Facultaire Saint-Luc-UCL, Brussels, Belgium and Ethics Committee Erasme Hospital, Brussels, Belgium. The patients/participants provided their written informed consent to participate in this study.

## Author Contributions

AG, SM, PB, and JBLPDW conceived the design of the study. AG and SM carried out all the experimental sessions from the recruitment to MSNA acquisition. AG, SM analyzed the data. DD and NM had full access to data and provided the statistical analyzes. SM, PB, JBLPDW, DD, and NM critically revised the manuscript. All the authors proofread and made corrections to this manuscript.

## Conflict of Interest

The authors declare that the research was conducted in the absence of any commercial or financial relationships that could be construed as a potential conflict of interest.

## Abbreviations

aLL-tVNS: auricular low-level transcutaneous vagus nerve stimulation
BF (bursts/min): number of bursts per minute
BMI (kg/m^2^): body mass index
DBP (mmHg): diastolic blood pressure
HF power (%): relative power of the high frequency band (0.15–0.4 Hz)
HF power (ms^2^): absolute power of the high frequency band
HR (beats/min): heart rate
LF power (%): relative power of the low frequency band (0.04–0.15 Hz)
LF power (ms^2^): absolute power of the low frequency band
LF/HF (%): ratio of the LF-to-HF power
MAP (mmHg): mean blood pressure
MSNA: muscle sympathetic nerve activity
MSNA activity (%): burst frequency multiplied by mean burst amplitude (AU) expressed in percentage of change from baseline value
RMSSD (ms): root mean square of the successive RR interval differences
RR (ms): time interval between two successive R-waves of the QRS
SBP (mmHg): systolic blood pressure
SDRR (ms): standard deviation of RR intervals.
