# Supplementary material for: Sympathetic Effect of Auricular Transcutaneous Vagus Nerve Stimulation on Healthy Subjects: A Crossover Controlled Clinical Trial Comparing Vagally Mediated and Active Control Stimulation Using Microneurography
Source: Front Physiol. 2020 Dec 3;11:599896. doi: 10.3389/fphys.2020.599896 (PMC7744823; doi:10.3389/fphys.2020.599896)
Supplement: Supplementary file 2 [file Table_1.pdf]

Supplemental data:

Additional table 1: Changes on HR, HRV, blood pressure and MSNA parameters

**Variable**	**n**	**Baseline**	**Stimulation**	**Recovery**
HR, bpm				
Active control (5Hz)	28	61.7 ± 8.4	60.6 ± 7.7	61.0 ± 7.4
aLL-tVNS-5Hz	28	61.3 ± 9.2	61.0 ± 8.7	61.5 ± 8.3
aLL-tVNS-20Hz	28	61.5 ± 8.3	61.4 ± 8.3	61.9 ± 8.0
RMSSD, ms				
Active control (5Hz)	28	47.7 ± 25.8	47.9 ± 24.8	48.5 ± 25.6
aLL-tVNS-5Hz	28	47.5 ± 25.4	48.4 ± 26.5	49.0 ± 27.6
aLL-tVNS-20Hz	28	46.4 ± 23.3	45.3 ± 23.7	47.8 ± 24.5
SDRR, ms				
Active control (5Hz)	28	70.5 ± 29.7	67.4 ± 25.7	73.7 ± 28.3
aLL-tVNS-5Hz	28	63.3 ± 22.4	66.9 ± 27.7	71.0 ± 25.3
aLL-tVNS-20Hz	28	64.3 ± 25.0	62.3 ± 24.7	72.0 ± 28.4
LF component, ms^2				
Active control (5Hz)	28	3120 ± 3269	2392 ± 2662	2340 ± 2299
aLL-tVNS-5Hz	28	2222 ± 2887	2616 ± 3303	2818 ± 2979
aLL-tVNS-20Hz	28	1880 ± 1784	2104 ± 2131	2655 ± 2732
HF component, ms^2				
Active control (5Hz)	28	1711 ± 2064	1541 ± 1760	1488 ± 1577
aLL-tVNS-5Hz	28	1789 ± 3201	1847 ± 3486	1891 ± 2421
aLL-tVNS-20Hz	28	1292 ± 1555	1416 ± 1630	1616 ± 1809
LF component, % power				
Active control (5Hz)	28	36.7 ± 17.8	33.1 ± 13.2	34.3 ± 12.7
aLL-tVNS-5Hz	28	35.9 ± 15.0	33.0 ± 13.9	32.7 ± 11.5
aLL-tVNS-20Hz	28	34.7 ± 14.0	34.1 ± 14.1	32.0 ± 14.4
HF component, % power				
Active control (5Hz)	28	20.5 ± 10.0	20.9 ± 9.3	20.8 ± 7.6
aLL-tVNS-5Hz	28	26.7 ± 11.2	21.2 ± 11.1	20.3 ± 9.9
aLL-tVNS-20Hz	28	21.8 ± 10.5	20.9 ± 9.1	18.9 ± 9.7
LF/HF ratio, % power ratio				
Active control (5Hz)	28	2.2 ± 1.4	1.8 ± 1.1	1.9 ± 1.0
aLL-tVNS-5Hz	28	1.6 ± 1.1	2.1 ± 1.5	2.0 ± 1.3
aLL-tVNS-20Hz	28	2.0 ± 1.5	2.0 ± 1.2	2.1 ± 1.5
SBP, mmHg				

Active control (5Hz)	28	115 ± 11	115 ± 12	114 ± 12
aLL-tVNS-5Hz	28	116 ± 11	115 ± 10	115 ± 10
aLL-tVNS-20Hz	28	115 ± 12	115 ± 10	115 ± 10
DBP, mmHg				
Active control (5Hz)	28	58 ± 9	58 ± 9	58 ± 10
aLL-tVNS-5Hz	28	59 ± 9	59 ± 9	59 ± 9
aLL-tVNS-20Hz	28	59 ± 9	59 ± 8	59 ± 9
MBP, mmHg				
Active control (5Hz)	28	77 ± 9	77 ± 10	77 ± 10
aLL-tVNS-5Hz	28	78 ± 9	77 ± 8	78 ± 9
aLL-tVNS-20Hz	28	77 ± 10	77 ± 8	77 ± 9
BF, bursts per minute				
Active control (5Hz)	16	17.6 ± 10.5	12.8 ± 10.1	16.1 ± 10.1
aLL-tVNS-5Hz	16	19.0 ± 10.5	14.7 ± 10.7	17.1 ± 9.8
aLL-tVNS-20Hz	15	16.1 ± 11.1	12.6 ± 11.8	17.9 ± 11.3
MSNA activity (%)				
Active control (5Hz)	16	100	89.4 ± 40.0	92.6 ± 27.5
aLL-tVNS-5Hz	16	100	81.3 ± 26.2	90.6 ± 25.6
aLL-tVNS-20Hz	15	100	87.3 ± 29.2	131.4 ± 29.5
